# Action control costs in task selection: Agents avoid actions with incompatible movement and effect features

**DOI:** 10.3758/s13414-024-02863-0

**Published:** 2024-03-21

**Authors:** Bence Neszmélyi, Roland Pfister

**Affiliations:** 1 Department of Psychology III, University of Würzburg, Röntgenring 11, 97070 Würzburg, Germany; 2 Department of Psychology, Trier University, Trier, Germany; 3 Institute for Cognitive and Affective Neuroscience (ICAN), Trier University, Trier, Germany

**Keywords:** Action control, Cognitive effort, Demand avoidance, Ideomotor theory, Action–effect compatibility

## Abstract

When a movement triggers effects with incompatible features, conflict between action and effect features creates costs for action planning and initiation. We investigated whether such action control costs also factor into action choices in terms of the principle of least effort. Participants completed a reaction-time experiment, where they were instructed to perform left and right mouse swipes in response to directional cues presented on the screen. Participants could select between two action options on each trial: Depending on which part of the screen (upper or lower) the action was performed in, the swipe resulted in a visual stimulus moving in the same (compatible) or in the opposite (incompatible) direction as the mouse. Incompatible action–effect mappings did indeed incur action control costs. In accordance with effort avoidance, the proportion of compatible choices was significantly above chance level, suggesting that action selection and initiation costs factor into participants preferences. Interestingly, however, participants’ choice tendencies were not predicted by the actual increase in action-initiation costs in the incompatible condition. This indicates that effort-related decisions are not simply based on monitoring performance in the actual task, but they are also influenced by preestablished notions of action-planning costs.

**Supplementary information:**

The online version contains supplementary material available at 10.3758/s13414-024-02863-0.

All things being equal, humans—and possibly all living organisms—prefer to select the easiest and least effortful means to reach a goal. This “law of less work” (Hull, 1943) or “principle of least action” (Maupertuis, 1750) has therefore been suggested to be a fundamental principle that governs choice behavior. It appears plausible that effort should determine action choices if different options come with clearly different physical investment. For example, choosing between jumping over a higher or lower hurdle (Cuvo et al., 1998) or walking a shorter or longer path (de Camp, 1920) yields clear preferences for the less effortful option. However, action choices are markedly different in settings known to trigger precrastination tendencies (Rosenbaum et al., 2014, 2019). In these experiments, participants were asked to carry one of two buckets to a finish line. One of the buckets was placed near the starting position whereas the other was placed a little further down the path. Here, participants routinely picked up the first bucket and happily carried it the extra mile to the finish line despite the increased effort. Although these results seemingly contradict the effort avoidance principle, the findings can be reconciled with this view when also considering cognitive effort: By starting the task at the first possible opportunity, participants save the effort of having to keep the task in memory. In accordance with these findings, several studies suggest that the tendency to minimize control costs and executive demands is an important factor in decision making (Botvinick & Rosen, 2009; Kool et al., 2010). Effort avoidance thus seems to take both cognitive and physical effort into account (Anderson, 1990; Baroody et al., 1986; Botvinick & Rosen, 2009; Kool & Botvinick, 2018; Kool et al., 2010).

While it seems evident that participants choose the less effortful alternative when agents are aware of differences in the costs associated with the action options, it is less clear whether choices are also affected when such differences are not obvious (Dunn et al., 2016; Gold et al., 2015). In the case of action planning and action initiation, different levels of effort might be required depending on the context in which actions are performed. However, due to the automatic nature of the processes involved in motor planning, compared with the examples in the previous paragraph, agents are arguably less aware of the differences in demands associated with the action options.

Ideomotor theorizing suggests that agents control their actions by anticipating action-contingent effects that they had previously associated to a motor movement (Harleß, 1861; James, 1890; see also Hommel, 2009; Kunde, 2001; Shin et al., 2010). These effects comprise what can be labelled an action goal, for example, the start of a video clip after clicking the play button with the computer mouse, as well as incidental effects such as the clicking sound of the mouse button or the proprioceptive and tactile sensations triggered by the action (Pfister, 2019). According to ideomotor theorizing, anticipating (i.e., mentally recollecting) sufficiently many of these effects will trigger the motor movement by activating previously learned associations between motor activity and following effects. This automatic activation of the associated movement plans is sometimes referred to as the *directive function* of the action effects (Eder et al., 2015; Ludwig et al., 2021).

The functional role of effect anticipation in action control is demonstrated most clearly in the action–effect compatibility paradigm (Kunde, 2001; Kunde et al., 2004; Pfister & Kunde, 2013; Shin & Proctor, 2012; Wirth et al., 2016). Here, participants respond to a stimulus and each of their actions elicits either compatible (e.g., left key press—effect on the left, right key press—effect on the right) or incompatible (e.g., left key press—effect on the right, right key press—effect on the left) action effects. Crucially, action effects always appear only after action execution so that any influence on performance measures can be attributed to anticipatory processes. The results usually show that actions with compatible effects are initiated faster, indicating that effect anticipations are functionally relevant for action selection and initiation (Kunde, 2001). More precisely, performance decrements with incompatible effects likely reflect conflict between features of body-related effects that code the actual movement and features of body-external events in the agent’s environment (Pfister & Kunde, 2013; for related evidence on the impact of imagined stimuli, see Ramsey et al., 2010; Tlauka & McKenna, 1998).

Behavioral effects related to action–effect incompatibility are a prime example of action control costs that can be reliably obtained in an experimental setting. The goal of the current study is to investigate whether these action control costs affect participants choices in a similar manner as costs associated with more salient examples of physical and mental effort. In addition to the directive function, studies investigating affective action consequences also attribute an *incentive function* to the effects (Eder et al., 2015; Ludwig et al., 2021), which refers to the ability of the effects to increase (or decrease) the attractivity of the associated action option (i.e., motivating agents to execute the action associated with the effect when competing alternatives are also available). One might assume that the directive and incentive functions are always aligned: If an action effect can easily activate the associated action (directive aspect) that action is executed effortlessly and according to the “law of less work,” it should be preferred over more costly alternatives (incentive aspect). The example of affective action consequences shows, however, that this is not always the case: Reaction times are reduced for actions that are expected to elicit effects with negative valence (compared with actions with neutral effects). Despite the lower cognitive cost indicated by the reduced initiation times, in a free choice task, agents still avoid the actions that lead to negative effects (Eder et al., 2015). Of course, in the case of affective stimuli, other factors are also at play. Still, these results raise the question whether an alignment between the directional and incentive function of action effects can be observed if other influences are controlled.

Against the backdrop of prior research, it is difficult to predict how cognitive costs in terms of selecting and initiating an action factor into participants preferences when selecting between various action options. One the one hand, it has been suggested that participants’ preferences are based on actual task demands. According to this approach, effort-based choices reflect the behavioral consequences of performing a cognitively demanding task (e.g., slower task execution; reduced performance on concurrent tasks). The soft constraints hypothesis (Gray, 2004; Gray & Boehm-Davis, 2000), for example, suggests that the cognitive system is sensitive to differences in action duration on the level of milliseconds, and it is these small differences that ultimately determine the effort associated with the action options, and thus determine choices. (Even if the agents are not consciously aware of that.) Approaches that presume a direct link between actual demands and participants’ decisions suggest that participants tend to select the less effortful alternative, even if differences between options are not salient and not recognized. For example, some studies indicate, that task switching is associated with costs that participants are motivated to avoid (Arrington & Logan, 2004; Botvinick & Rosen, 2009; Kool et al., 2010): When people are asked to choose between two decks of cards, and one of the decks is associated with more frequent task-switching, participants tend to choose the other deck more often, even if they are not aware of the difference between the two decks. According to this approach, we would expect that the extra effort that is required for dealing with conflicting sensory information when performing actions with incompatible effects would result in the avoidance of this action option in a free choice task. Furthermore, a correlation would be expected between measures reflecting effort and measures that quantify the avoidance of actions with incompatible effects.

On the other hand, it seems that in many instances, participants’ choices cannot be predicted based on the behavioral correlates of cognitive effort. In such cases, action choices seem surprisingly indifferent to the actual effort required by the task (e.g., Dunn et al., 2016; Gold et al., 2015). Using the same card selection task as previous work (Kool et al., 2010), more recent studies only found an avoidance of the deck that required frequent task switching if participants were made aware of the difference between the two decks (Gold et al., 2015). Furthermore, healthy controls showed more pronounced effort avoidance than people with schizophrenia in this work. This also supports the idea that effort awareness and the explicit construal of the task plays a key role in establishing effort-related preferences, since actual switching-related behavioral costs were higher for patients than for healthy participants, while the control group was better at detecting differences in the required effort. Similar conclusions regarding the role of perceived and actual effort come from a study using a reading task (Dunn et al., 2016). When task difficulty was manipulated by rotating reading direction and letter orientation, participants’ preferences were determined by the perceived difficulty of the options and not by the actual demand—as reflected in behavioral and physiological measures. If participants choices are not determined by actual task demands but by perceived task difficulty, then preference of actions with compatible effects is not warranted—although still possible if perceived difficulty coincides with (or reflects) actual demands. In this case, we also would not expect a correlation between the behavioral measures related to conflict resolution costs and participants’ preference of actions with compatible effects.

To investigate this question, we devised an experimental setting where participants could freely decide whether to perform actions with compatible or with incompatible visual effects. More precisely, participants had to perform left and right mouse swipes in response to directional cues. Their responses elicited visual effects that were irrelevant for deciding on the direction of the response. Before each response, however, they could select in which part of the screen (top, bottom) they wanted to perform the task. In one location, movement of an object on the screen followed the direction of the executed action. In the other location, the object always moved in the opposite direction as the movement performed by the participant. Effort avoidance would occur as a preference for the option with the compatible effect as compared with the incompatible effect. If participants were to minimize effort, we further planned to investigate whether this tendency was related to the actual size of the cost as measured via initiation times of their responses. We therefore tested (a) whether participants chose the compatible option at above-chance frequencies and (b) whether participants with a more pronounced reaction time advantage for the compatible option would also have a stronger inclination to select the compatible option over the incompatible one.

## Method

### Participants

Power analysis indicated that comparing the share of compatible choices to chance level with a one-sample *t* test would require 51 participants to reveal an effect of the magnitude *d* = 0.4 at alpha level 0.05 and with 1 − β = 0.8 power. Following the suggestion of Brysbaert (2019), we selected this target effect size because it can be regarded as a good estimate for the effect size of smallest interest in psychological research. But the selection is also in accordance with research on effort avoidance and with studies investigating response–effect compatibility: Previous experiments that used choice proportion as a dependent variable to investigate effort avoidance, usually reported medium to large effect sizes. Using task switching to manipulate the level of effort, Gold et al., (2015, Experiment 3) observed an effort avoidance effect of *d*_z_ = 0.69 for healthy participants and *d*_z_ = 0.47 for people with schizophrenia. With a reading task where the manipulation of reading direction and letter orientation was used to operationalize task demands, Dunn et al. (2016) found effect sizes (*d*) between 0.87 and 2.28 when both the actual and the perceived demands differed substantially between action options. Similarly, response–effect compatibility effects with left–right movements and dynamic action effects yielded medium to large effects throughout (e.g., *d*_z_ = 0.48 in Schwarz et al., 2018, and *d*_z_ = 0.63 in Liesner et al., 2020).

We therefore recruited participants on the Prolific website until we had a final, counterbalanced sample of 54 participants who did not show signs of strong strategy use for their choices (see the preregistration at https://aspredicted.org/5pb8r.pdf; mean age = 24.611 years, age range: 19–30 years, male: 21, female: 33, all right-handed). This sample size makes it possible to reveal and effect size of *d* = 0.39 (at alpha level 0.05 and with 1 − β = 0.8 power). A total of 93 participants were tested. Analysis on the full sample is available in the Supplementary Material. Participants received £2.50 for completing the experiment. Before starting the experimental task, participants were informed about the study, and they agreed to the terms and conditions of participation.

### Stimuli

Since the experiment was conducted online, all stimuli were scaled relative to the participant’s individual screen size to enable a comparable setup across devices, with 1 height unit corresponding to the full height of the screen. Each trial began with a hand icon and two target objects, a blue and a green wagon, displayed on screen against a grey background. The hand icon was 0.04 × 0.04 height units and it was presented in the center of the screen. The two wagons were 0.24 × 0.11 height units and were presented at the vertical midline of the screen, 0.4 height units above and below the hand icon. Two white vertical lines (width: 0.02 height unit, height: 1 height unit) were presented 0.65 height units left and right of the vertical midline.

### Task

**Fig. 1:**
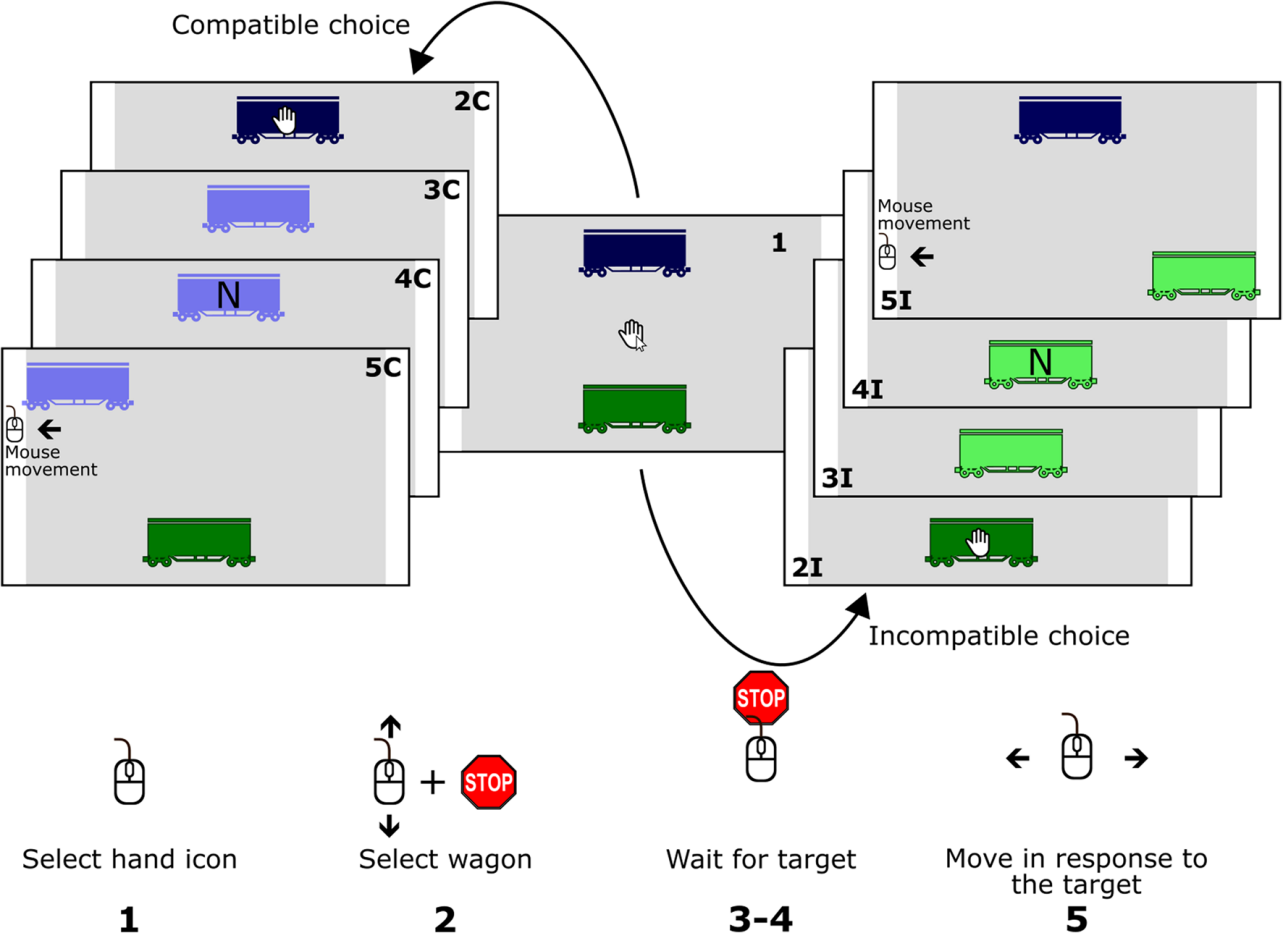
The wagon selection task. Participants were instructed to select one of the two wagons on the screen, one of which followed the mouse movements (left: 2C–5C; compatible response–effect mapping), while the other moved in the opposite direction (right: 2I–5I; incompatible response–effect mapping). On the bottom of the figure, the mouse movements that participants had to perform to complete a trial are displayed. (Color figure online)

Figure 1 shows a schematic of the experimental setup and procedure. In each trial, participants’ task was to select one of the wagons and then perform a left or right mouse swipe according to the visual cue presented on the screen. The mouse swipe was immediately translated into a movement of the wagon towards one of the white lines. One of the wagons moved in the same direction as the mouse swipe (compatible movement), while the other moved in the opposite direction (incompatible movement). The location of the blue and green wagons (top and bottom of the screen) and the assignment of compatible and incompatible movement to the wagons was randomized across participants. The location and movement assignments were constant for each individual session. Before the start of the experiment, participants were informed about how their movements would affect the movement of the wagons.

Participants were instructed to move the mouse cursor over the hand icon after the trial started and to pick it up. The cursor disappeared upon left-clicking the hand icon, and the hand icon now followed the participant’s mouse movements. Participants could select one of the wagons by moving the hand icon over the object and then holding still for 2 s. If they did that, the selection of the wagon was indicated by the color of the wagon turning brighter. At the same time, the hand icon disappeared. After a random delay of 1–3 s, a cue appeared (the letter *N* or *Z*), signaling the required mouse swipe for the participant. The assignment of the letters to the required movement direction was randomized across participants. When participants executed the mouse swipe, the movement of the wagon (direction, speed) was determined by the horizontal component of the mouse movement. That is, the wagon could only move left and right but not in a vertical direction. The selected wagon moved either in the same (compatible movement) or in the opposite (incompatible movement) direction as the mouse. Participants were instructed to keep moving until the wagon reached one of the white lines. At the line, the wagon stopped, even if the movement was continued.

The direction cues (letters *N* and *Z*) were displayed for 2 s. If the swipe was not started within this time frame, participants received an error message at the end of the trial. An error message was also displayed if participants swiped in the wrong direction. If participants started the movement before the direction cue appeared, the wagon turned back to a darker color and the hand icon reappeared. In this case, participants had to re-select the wagon to continue with the trial. After finishing the movement, the wagons remained on the screen for 2 s, but participants could no longer move them. Before the start of the next trial, all stimuli disappeared from the screen for 1 s, except for the two white lines.

### Procedure

Instructions were followed by a practice phase. This phase began with only the upper wagon being displayed on the screen. Participants had to select and move this wagon as described above. The movement assignment of the wagon was the same as later, during the experimental phase. Detailed instructions were displayed on the screen throughout the practice phase. If participants deviated from the instructions, an error message was displayed, and if participants finished the trial with a swipe in the wrong direction, the practice phase was repeated. This demonstration was repeated with the wagon at the bottom of the screen. Participants then completed a short block of 10 trials with both wagons on the screen and were instructed to select both of them a few times during the block.

After the practice phase, participants completed four experimental blocks with 25 trials each. Participants were given the instruction that in each trial they could freely select the wagon they would like to move. They were only asked to select each of the wagons at least occasionally within each block. Between the blocks, participants were given the opportunity to take a short break and they could start the next block at leisure.

### Data acquisition

The study was conducted online. The experiment was written in PsychoJS and it was hosted on the Pavlovia website. Participants were asked to use an external mouse during the experiment. During each trial, we recorded selection times (from displaying the action options until the selection of the wagon^1^), selected option (compatible or incompatible wagon), reaction times (time elapsed between the presentation of the directional cue and the start of the movement—defined as the time point when the wagon was first moved 0.05 height units from the starting position), and movement times (time elapsed between the start of the movement and the moment when the wagon stopped at the white line). Due to limitations of the PsychoJS software, the sampling rate of the mouse movements corresponded to the refresh rate of the participant’s monitor. We also recorded whether the correct action was executed. If participants moved in the wrong direction, the trial was categorized as an error trial.

### Statistical analysis

The variable of main interest was the proportion of compatible choices. We therefore computed the proportion for each participant and compared the mean proportions to chance level (0.50) with a one-sample *t* test. It takes some time until action–effect associations are established (Wolfensteller & Ruge, 2011) and cognitive costs associated with the compatible and incompatible options are assessed. Thus, it is possible that choice preferences only emerge after longer experience with the task. To account for this possibility, choice proportions were also assessed separately in each block. The block-wise choice proportions were submitted to a one-way repeated-measures ANOVA (with block as the within-subjects factor), and in each block the proportion of compatible choices was compared with chance level. For each participant and movement option, we further computed the error percentage, mean selection time, reaction time and movement time. The four measures were submitted to paired *t* tests to compare the compatible and the incompatible response–effect mapping. We also assessed the relationship between choice proportion and incompatibility-related action costs: Reaction time deficits (i.e., the reaction time difference between compatible and incompatible trials) and accuracy deficits (i.e., the difference in error percentage between compatible and incompatible trials) were correlated with choice proportion. It is important to note that such correlations do not reveal the causal link between motor control costs and action preference: A significant positive correlation could suggest that difficulty of action initiation was considered when a choice between action options had to be made, but it could also indicate that more frequent selection of the compatible option resulted in more experience with compatible action effects, which in turn lead to faster action initiation times.

Since error percentage was marginally lower in compatible trials (see Results), the higher frequency of compatible choices could possibly also be explained by a tendency to switch choices after errors (without considering the action–effect relation of the following choice). Thus, we also compared the percentage of compatible choices to chance level in the subset of participants who had an error rate (percentage of errors) in incompatible trials that was either smaller or equal to their error rate in compatible trials. We also compared compatible choice proportion between this subsample and the remaining part of the final sample (i.e., participants with a higher error rate in incompatible trials than in compatible trials).

We excluded participants from the analyses who consistently selected the same wagon throughout a whole block and those who had less than 10 trials remaining for either of the action options after removing erroneous actions (throughout the whole experiment). We also excluded participants who applied a strategy of systematically alternating between the two action options (option change in more than 90% of the trials) and who exceeded the reaction time limit (2 s) or performed a swipe in the wrong direction on more than 30% of the trials.^2^ Participants who used a monitor with a frame rate below 50 Hz were also not included in the analyses. (Since the sampling frequency of the program was tied to the refresh rate of the monitor, low sampling frequency is associated with a reduced accuracy in the recording of movement parameters. Furthermore, smoothness of the object motion on the screen also depends on the monitor refreshment rate.) The analysis of choice proportions, error rates and selection times included all trials. During the analysis of reaction times and movement durations, however, trials were rejected if participants had reaction times that exceeded the time limit, or if they executed a swipe in the wrong direction. Also, we applied *z*-score-based outlier rejection to selection times, reaction times, and movement times. Trials with *z* scores exceeding an absolute value of 2.5 (calculated separately for each participant, and selection option) were excluded from the analyses.

All statistical analyses were conducted in R (Version 3.6.3; R Core Team, 2020). We used the packages ez (Version 4.4.0; Lawrence, 2016), lme4 (Version 1.1.23; Bates et al., 2015), schoRsch (Version 1.9.1; Pfister & Janczyk, 2020), and tidyverse (Version 1.3.0; Wickham et al., 2019) for data management and statistical analyses, and the packages ggplot2 (Version 3.3.3; Wickham, 2016), and sjPlot (Version 2.8.10; Lüdecke, 2021) for data visualization.

## Results

**Fig. 2:**
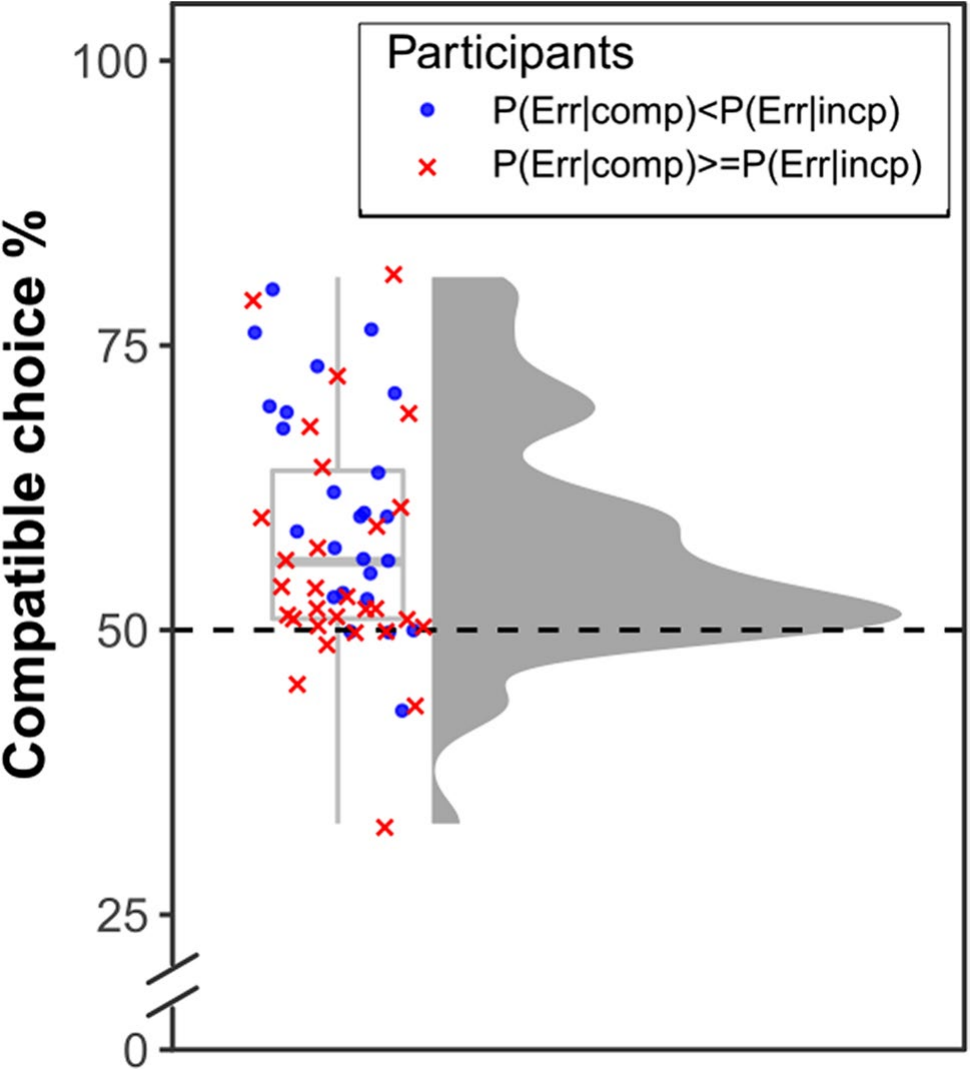
Percentage of compatible choices for the participants included in the final sample. Points and crosses represent individual choice percentages of participants, accompanied by a boxplot showing the median (thick line), the interquartile range (IQR; box) and the 1.5 IQR interval from the upper and lower limits of the IQR (whiskers). Participants who had a higher error proportion in the incompatible condition are indicated by blue dots, participants who committed more errors in the compatible condition, or an equal number of errors in both conditions, are indicated by red crosses. The grey area shows a kernel density estimate for the distribution of the individual values. (Color figure online)

Figure 2 shows the distribution of choice percentages across participants. Participants chose the compatible option on 58.2% (*SD* = 10.3%) of the trials, and this percentage clearly exceeded chance level, *t*(53) = 5.83,* p* < 0.001, *d* = 0.79. The above-chance preference for compatible choices was stable across the experiment as indicated by a nonsignificant effect of block in a repeated-measures analysis of variance (ANOVA), *F*(3, 159) = 0.28, *p* = 0.839, η_p_^2^ = 0.01, while individual tests per block were significant throughout (Table 1).

**Table 1:** Percentage of compatible choices in the four experimental blocks

	*M* (%)	*SD* (%)	Comparison to chance level^1^
1^st^ block	58.15	11.44	5.23*** *0.71*
2^nd^ block	57.93	11.68	4.99*** *0.68*
3^rd^ block	57.70	11.49	4.93*** *0.67*
4^th^ block	58.89	13.33	4.90*** *0.67*

*** *p* < 0.001

^1^We report *t* values with significance levels and effect sizes (in italics: Cohen’s *d*_z_)

Table 2 summarizes all performance measures as a function of action–effect compatibility (see Table 3 for the effective trial number for each analysis). Error percentage was marginally higher in the incompatible compared with the compatible trials, *t*(53) = 1.70, *p* = 0.095, *d*_z_ = 0.23. The correlation between compatible choice percentage and the difference of error percentages (error percentage in the incompatible trials minus error percentage in the compatible trials) was not significant, *r*(52) = 0.21, *p* = 0.138. Selection times did not differ significantly between compatible and incompatible choices, either for the complete selection phase, *t*(53) = 1.53, *p* = 0.133, *d*_*z*_ = 0.17, or for the cursor movement phase, *t*(53) = 0.22, *p* = 0.824, *d*_*z*_ = 0.03. Analysis of reaction times indicated that participants initiated the movements faster in compatible (*M* = 679 ms, *SD* = 181 ms) than in incompatible trials (*M* = 708 ms, *SD* = 196 ms), *t*(53) = 2.96, *p* = 0.005, *d*_*z*_ = 0.40. No correlation was observed between compatible choice percentage and reaction time advantage for compatible trials, *r*(52) = 0.138, *p* = 0.320. Movement times did not differ significantly between compatibility conditions, *t*(53) = 0.71, *p* = 0.480, *d*_z_ = 0.10.

**Table 2:**
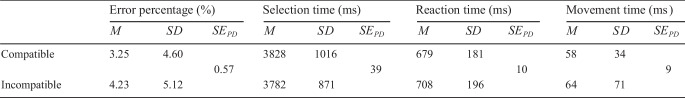
Error percentage, selection time, reaction time, and movement time for compatible and incompatible trials

Each pair of means is accompanied by the corresponding standard error of paired differences (*SE*_*PD*_; Pfister & Janczyk, 2013)

**Table 3:** Number of trials used for the reported analyses

	Choice percentage and error percentage		Selection time		Reaction time and action duration		GLMM for choice		GLMM for RT	
	*M*	*SD*	*M*	*SD*	*M*	*SD*	*M*	*SD*	*M*	*SD*
Compatible	58.17	10.30	56.46	9.85	53.65	9.96	55.46	9.70	50.44	10.18
Incompatible	41.83	10.30	40.72	10.08	38.37	9.70	40.54	9.70	36.89	9.35

In the final sample of 54 participants, there were 20 participants who had a higher error rate in the compatible condition, nine participants who had identical error rates in the two conditions, and 25 participants who committed errors at a higher rate in the incompatible condition. In the subsample consisting of participants with equal error rates in the two conditions or higher error rate in the compatible condition, a similar preference of the compatible option emerged as in the final sample, *t*(28) = 2.99, *p* = 0.006, *d*_*z*_ = 0.56. It has to be noted, however, that percentage of compatible choices in this subset was marginally different, *t*(52) = 1.89, *p* = 0.064, *d* = 0.52,^3^ from compatible choice percentage in the other part of the sample (i.e., participants who committed errors at a higher rate in the incompatible condition), where the preference for the compatible option was slightly more pronounced, *t*(24) = 5.66, *p* < 0.001, *d*_z_ = 1.13 (Table 4).

**Table 4:** Number of errors and error percentage in the subgroups based on error percentage in compatible and incompatible trials

	Group size		Comp. choice (%)	Number of errors		Percentage of errors	
				Comp	Incomp	Comp	Incomp
Error percentage in compatible trials ≥ error percentage in incompatible trials	29	*M*	55.76	2.24	1.03	4.06	2.31
		*SD*	10.37	2.01	1.64	5.31	3.67
Error percentage in incompatible trials > error percentage in compatible trials	25	*M*	60.96	1.40 ±	2.52	2.32	6.45
		*SD*	9.68	2.08	2.50	3.49	5.70

## Discussion

When presented with a choice between actions with compatible and incompatible effects, participants showed a clear preference for the compatible option, which was remarkably consistent across participants. The finding that participants preferred the action option characterized by less demand during action initiation indicates that effort related to action planning and initiation directly feeds into decisions about which context to choose for upcoming actions. However, the fact that we did not observe a connection between participants’ choices and the eventual amount of conflict experienced when actually performing an action with incompatible effects indicates that a more nuanced interpretation of the results is required: At first glance, it seems at odds with the effort-based explanation, that it was not the participants who showed the largest compatibility-related motor costs that exhibited the strongest tendency to avoid the incompatible option. For a better understanding of the above results, two questions have to be addressed: (1) what constitutes the effort that participants aim to avoid when they choose the compatible option and (2) how expectations of effort are generated. Although a definitive answer to these questions cannot be derived from the current data set, the analyses we performed provide several hints regarding the interpretation of the results.

### Motor costs

The concept of the study followed from the assumption that the difference in effort associated with the compatible and incompatible option was based on costs related to planning and initiating an action in the face of conflicting action and effect features in the incompatible condition. Such costs were observed in numerous studies (Kunde, 2001; Kunde et al., 2004; Pfister & Kunde, 2013; Wirth et al., 2016), but the question of how this affects the agents’ choices was less explored. We are only aware of one study that addressed this topic: Richardson et al. (2020) reported that in a free-choice task participants showed a tendency to avoid action options where motor planning and initiation were hampered by partial repetition costs (slower initiation times for actions that overlap versus do not overlap with another action). In the study of Richardson and colleagues, avoiding partial overlap helps both planning a new movement and retaining a previously established motor plan—thus, it is not evident if it is indeed the effort related to action planning that determines participants preferences. By using a different paradigm that focuses on the role of effect anticipation in action planning, our results confirm that motor planning costs influence choice preferences even though in common experimental settings these costs only come on a scale of milliseconds.

However, besides motor planning costs, there might also be other costs associated with the incompatible condition that could have had an influence on participants choices: In trials where participants selected the incompatible option, the effects were characterized by a different spatial feature than the one associated with the directional cue (i.e., letters *N* and *Z*). For example, if a left directional cue was presented, the effect of the corresponding response was the rightward movement of the wagon. The conflicting spatial features associated with the directional cue and with the visual effect can make the interpretation of error feedback and the correction of errors more difficult in the incompatible condition. Thus, the preference of the compatible option could also be explained by the cost of errors being perceived as higher in incompatible trials. However, this alternative account was not supported by the analysis of responses before the first error (see the Supplementary Material). We are therefore confident that the observed preference for contexts with compatible action–effect mapping is indeed a function of the effort required to plan and initiate an action.

### Expected effort

The fact that participants showed a tendency to avoid the more effortful action option indicates that they established expectations regarding the demands associated with each option. The most obvious source of such expectations could be participants’ monitoring and evaluation of actual performance measures or of cognitive processes that are linked to such performance measures. For example, noticing that errors are more frequent or response times slower in a certain task could lead to those tasks being perceived as difficult (Gray, 2004; Gray & Boehm-Davis, 2000; Kool et al., 2010). Error rates were indeed higher and responses slower for the incompatible action option in the current study. However, the fact that on the individual level, effort avoidance tendencies were not determined by the degree for conflict experienced (i.e., no correlation between the magnitude of the compatibility effect and the proportion of compatible choices) reduces the likelihood of a direct link between experienced effort and choice preference.

An explanation for the lack of correlation between performance measures and choice proportion might be that the influence of actual compatibility costs is mediated by effort sensibility. That is, how well actual costs are translated into expected effort might depend on the ability of participants to detect differences in task demands. A similar idea has been proposed by Gold et al. (2015), which provided a good explanation for reduced effort avoidance in people with schizophrenia: Although the actual costs induced by the demand manipulation were larger in patients than in healthy controls, they showed deficiency in their ability to recognize differences in demand. However, in the current study, a significantly higher proportion of the compatible choices was also observed for the subsample that made more errors when choosing the compatible action option (or equal number of errors with both effect types),^4^ which reduces the plausibility of actual performance measures influencing participants choices, even when considering mediation by effort sensitivity.

A second and not mutually exclusive explanation is that experience about more fluent action control with compatible effects is acquired prior to the experiment, during everyday interactions with the environment, and the observed choice tendencies reflect the generalization of such experiences to the experimental context. This assumption would explain the weak link between performance measures and action selection. It would also account for why the preference of actions with compatible effects could already be observed in the first experimental block. Action–effect associations are established within a few trials (Gouret & Pfeuffer, 2021; Wolfensteller & Ruge, 2011). It is difficult to measure, however, how fast compatibility-related differences in RTs appear, and after that, how long it takes until agents assess the costs associated with the action options. A study that investigated compatibility effects in a changing context did not find a significant difference in RTs between actions with compatible and incompatible effects during the first 12 trials (Gouret & Pfeuffer, 2021). Thus, the fact that, in the current study, a similar choice proportion was found in the first block as after more extensive experience might suggest that the preference for the compatible option has been established before participants could reliably evaluate their own performance on the actual task.

Even if the actual differences in control costs (as measured by RT and error rate differences in the compatible and incompatible conditions) do not influence preferences, based on an influence in the opposite direction, one would still expect a correlation between these two measures: RTs and error rates should decrease with more exposure to a task, and thus choice proportions of the compatible versus incompatible options should impact RT and error rate differences. The lack of the correlation could be explained by the fact that the compatible choice rate was between 50–60% for a substantial proportion of the participants, and such small imbalance might not be sufficient to cause an experience-based effect. This idea is supported by the observation that in the extended sample (where participants with more extreme choice proportions are also represented), the correlations are somewhat stronger (see the Supplementary Material).

### Alternative explanations

The explanations discussed until this point all suggest that participants’ preference of the compatible option was determined by their goal of avoiding unnecessary effort. There are alternative accounts, however, that do not draw on effort avoidance. First, participants might be expected to switch options in response to errors. If participants tend to switch to a different option after committing an error, higher error frequency in the incompatible condition could result in the observed choice patterns (without requiring any anticipatory processes). Additional analyses, however, did not support this explanation (see the Supplementary Material).

Second, choice might not be based on effort during action planning and initiation (at least not directly), but on the degree of control participants felt when performing the actions: It is possible that they felt more in control of the action effects when these were compatible with the actions (Ebert & Wegner, 2010; Farrer et al., 2008; Potts & Carlson, 2019). This increased feeling of control could be based both on retrospective considerations (e.g., evaluating the relationship between effector and effect movement after the action has been performed) or actually induced by the fluency of action selection (Chambon & Haggard, 2012; Sidarus et al., 2013, 2017). Control over the environment has been suggested to be a strong motivator (Higgins, 2012; Karsh & Eitam, 2015b). According to the *control-based response selection framework* (Karsh & Eitam, 2015b), preference of the action option with a higher level of control explains the choice of actions with contingent body-external effects over actions without such effects and over actions that are characterized by reduced action–effect contingency (Hemed et al., 2020; Karsh & Eitam, 2015a). Similar explanations have also been suggested for the preference of actions with immediate effects over actions with delayed consequences (Karsh et al., 2016). The tendency to select actions with compatible effects over actions with incompatible effects would fit well into this pattern, even though both situations came with perfect contingency.

An explanation based on Gestalt principles is also plausible: Visual and tactile/proprioceptive effects moving in the same direction could contribute to them being easier to integrate into a unified percept (Gestalt) while their movement into opposite directions could limit Gestalt-based perception, leading to a preference of the compatible condition. This explanation, however, can also be reconciled with the effort-based account: Gestalt principles have been also suggested to play a role in action control (see Klapp & Jagacinski, 2011, for a review). In particular, bimanual coordination has been shown to be more effective if the action effects comply with Gestalt principles (Kovacs et al., 2010; Mechsner et al., 2001), but such findings might generalize to other aspects of action control. The assumption that would follow from this reasoning (control is less effortful if the action–effects can be integrated into a Gestalt), is similar to the argument that we made for differences in effort when performing actions with compatible and incompatible effects (see also Klapp & Jagacinski, 2011, for an explanation that connects the ideomotor and Gestalt-based frameworks).

In the current study, we did not examine participants subjective assessment of the action options. As a consequence, we cannot determine whether choice preferences were also reflected in a conscious positive or negative evaluation of the two options, and we do not know what participants themselves perceived as the motivation for their choices. Examining this in future studies could contribute to a better understanding of processes that result in the preference of the action option associated with smaller motor control costs.

### Conclusion

Ideomotor studies on human action control consistently showed that actions are initiated faster if they elicit compatible rather than incompatible sensory effects. The current study shows that compatibility-related differences in the efficiency of action control processes are directly reflected in the preferences of the agents for different action contexts: When participants are presented with a choice between an action with compatible and an action with an incompatible effect, participants are more likely to select the former. This preference may take into account the experience of effort for individual actions and it might likewise reflect preestablished assessments of action planning effort, and the impact may be mediated by feelings or judgments of agency. The findings reported here open the door for studies that could examine how agents’ knowledge about, or experience with, action control in different settings might influence the choices that they face while interacting with their environment. Future experiments should further assess how the observed impact of action-control-related effort depends either on conscious strategies or on implicit processes during action monitoring.

### Supplementary information

Below is the link to the electronic supplementary material.

Supplementary file1 (DOCX 218 KB)

## Data Availability

Data collected in the study are available on the Open Science Framework (https://osf.io/zxtpa/).

## Footnotes

1 We also assessed the movement phase of the selection times separately: This included the interval between picking up the hand icon and selecting the wagon.

2 In the current experiment, participants might have interpreted the instruction as suggesting a specific strategy (e.g., systematically switching between options). Since previous findings indicate that the influence of effort sensitivity can be masked by several other factors, like preference of certain stimulus patterns and locations, or strategies that participants apply to make the task less monotonous (Gold et al., 2015), we excluded participants who seemed to adopt a systematic non-effort-related strategy. The Supplementary Material includes detailed analyses with less stringent rejection criteria. The central findings reported in the main text were fully supported by analyses based on this larger sample.

3 Beside testing whether controlling the influence of post-error-switching affects our main findings, this comparison can be also used to assess the relationship between incompatibility-related motor cost (as indicated by errors) and preference of the compatible option. This is, however, the same question that is also assessed by the correlation between the difference in error percentages and the percentage of compatible choices. Thus, it is more adequate to interpret the results of this *t* test at an adjusted significance threshold (alpha = .025).

4 It also has to be noted, however, that the subsample with higher error rates in the incompatible condition showed marginally larger preference for compatible actions than participants who made more errors when choosing the compatible action option (or equal number of errors with both effect types).

## References

[CR1] Anderson JR (1990). The adaptive character of thought.

[CR2] Arrington CM, Logan GD (2004). The cost of a voluntary task switch. Psychological Science.

[CR3] Barlas, Z., & Kopp, S. (2018). Action choice and outcome congruency independently affect intentional binding and feeling of control judgments. *Frontiers in Human Neuroscience*, *12*, Article 137. 10.3389/fnhum.2018.00137 10.3389/fnhum.2018.00137 PMC5904194 29695958

[CR4] Baroody AJ, Ginsburg HP, Hiebert J (1986). The relationship between initial meaningful and mechanical knowledge of arithmetic. Conceptual and procedural knowledge: The case of mathematics.

[CR5] Bates, D., Maechler, M., Bolker, B., & Walker, S. (2015). Fitting linear mixed-effects models using lme4. *Journal of Statistical Software, 67*(1), 1–48. 10.18637/jss.v067.i01

[CR6] Botvinick MM, Rosen ZB (2009). Anticipation of cognitive demand during decision-making. Psychological Research Psychologische Forschung.

[CR7] Brysbaert, M. (2019). How many participants do we have to include in properly powered experiments? A tutorial of power analysis with reference tables. *Journal of Cognition*, *2*(1), Article 16. 10.5334/joc.72 10.5334/joc.72 PMC6640316 31517234

[CR8] Chambon V, Haggard P (2012). Sense of control depends on fluency of action selection, not motor performance. Cognition.

[CR9] Cuvo AJ, Lerch LJ, Leurquin DA, Gaffaney TJ, Poppen RL (1998). Response allocation to concurrent fixed-ratio reinforcement schedules with work requirements by adults with mental retardation and typical preschool children. Journal of Applied Behavior Analysis.

[CR10] De Camp JE (1920). Relative distance as a factor in the white rat’s selection of a path. Psychobiology.

[CR11] Dunn TL, Lutes DJC, Risko EF (2016). Metacognitive evaluation in the avoidance of demand. Journal of Experimental Psychology: Human Perception and Performance.

[CR12] Ebert JP, Wegner DM (2010). Time warp: Authorship shapes the perceived timing of actions and events. Consciousness and Cognition.

[CR13] Eder AB, Rothermund K, De Houwer J, Hommel B (2015). Directive and incentive functions of affective action consequences: An ideomotor approach. Psychological Research Psychologische Forschung.

[CR14] Engström, M., Karlsson, T., Landtblom, A.-M., & Craig, A. D. (2015). Evidence of conjoint activation of the anterior insular and cingulate cortices during effortful tasks. *Frontiers in Human Neuroscience*, *8*. 10.3389/fnhum.2014.01071 10.3389/fnhum.2014.01071 PMC4306292 25674057

[CR15] Farrer C, Bouchereau M, Jeannerod M, Franck N (2008). Effect of distorted visual feedback on the sense of agency. Behavioural Neurology.

[CR16] Gold JM, Kool W, Botvinick MM, Hubzin L, August S, Waltz JA (2015). Cognitive effort avoidance and detection in people with schizophrenia. Cognitive, Affective, & Behavioral Neuroscience.

[CR17] Gouret F, Pfeuffer CU (2021). Learning to expect and monitor the future: How fast do anticipatory saccades toward future action consequences emerge?. Journal of Experimental Psychology: Human Perception and Performance.

[CR18] Gray W (2004). Soft constraints in interactive behavior: The case of ignoring perfect knowledge in-the-world for imperfect knowledge in-the-head. Cognitive Science.

[CR19] Gray WD, Boehm-Davis DA (2000). Milliseconds matter: An introduction to microstrategies and to their use in describing and predicting interactive behavior. Journal of Experimental Psychology: Applied.

[CR20] Harleß E (1861). Der Apparat des Willens [The apparatus of will]. Zeitschrift Für Philosophie Und Philosophische Kritik.

[CR21] Hemed E, Bakbani-Elkayam S, Teodorescu AR, Yona L, Eitam B (2020). Evaluation of an action’s effectiveness by the motor system in a dynamic environment. Journal of Experimental Psychology: General.

[CR22] Higgins, E. T. (2012). *Beyond pleasure and pain: How motivation works*. Oxford University Press.

[CR23] Hommel B (2009). Action control according to TEC (theory of event coding). Psychological Research/ Psychologische Forschung.

[CR24] Hull, C. L. (1943). *Principles of behavior.* Appleton-Century.

[CR25] James, W. (1890). *The principles of psychology* (Vol. 2). Harvard University Press.

[CR26] Karsh N, Eitam B (2015). I control therefore I do: Judgments of agency influence action selection. Cognition.

[CR27] Karsh, N., & Eitam, B. (2015b). Motivation from control: A response selection framework. In P. Haggard & B. Eitam (Eds.), *The sense of agency*. Oxford University Press. 10.1093/acprof:oso/9780190267278.003.0012

[CR28] Karsh N, Eitam B, Mark I, Higgins ET (2016). Bootstrapping agency: How control-relevant information affects motivation. Journal of Experimental Psychology: General.

[CR29] Klapp ST, Jagacinski RJ (2011). Gestalt principles in the control of motor action. Psychological Bulletin.

[CR30] Kool W, Botvinick M (2018). Mental labour. *Nature Human*. Behaviour.

[CR31] Kool W, McGuire JT, Rosen ZB, Botvinick MM (2010). Decision making and the avoidance of cognitive demand. Journal of Experimental Psychology: General.

[CR32] Kovacs AJ, Buchanan JJ, Shea CH (2010). Impossible is nothing: 5:3 and 4:3 multi-frequency bimanual coordination. Experimental Brain Research.

[CR33] Kunde W (2001). Response–effect compatibility in manual choice reaction tasks. Journal of Experimental Psychology: Human Perception and Performance.

[CR34] Kunde W, Koch I, Hoffmann J (2004). Anticipated action effects affect the selection, initiation, and execution of actions. The Quarterly Journal of Experimental Psychology Section A.

[CR35] Lawrence, M. A. (2016). *ez: Easy analysis and visualization of factorial experiments* (R Package Version 4.4–0) [Statistical software]. Retrieved from https://CRAN.R-project.org/package=ez. Accessed 2 Nov 2022.

[CR36] Liesner, M., Kirsch, W., & Kunde, W. (2020). The interplay of predictive and postdictive components of experienced selfhood. *Consciousness and Cognition*, *77*, Article 102850. 10.1016/j.concog.2019.102850 10.1016/j.concog.2019.102850 31731032

[CR37] Ludwig, J., Dignath, D., & Lukas, S. (2021). Positive and negative action–effects improve task-switching performance. *Acta Psychologica*, *221*, Article 103440. 10.1016/j.actpsy.2021.103440 10.1016/j.actpsy.2021.103440 34717256

[CR38] Lüdecke, D. (2021). *sjPlot: Data visualization for statistics in social science* (R Package Version 2.8.10) [Statistical software]. Retrieved from https://CRAN.R-project.org/package=sjPlot. Accessed 2 Nov 2022.

[CR39] Maupertuis, de P. L. M. (1750). *Essai cosmologie (Essay on cosmology)*. Author.

[CR40] Mechsner F, Kerzel D, Knoblich G, Prinz W (2001). Perceptual basis of bimanual coordination. Nature.

[CR41] McGuire, J. T., & Botvinick, M. M. (2010). The impact of anticipated cognitive demand on attention and behavioral choice. In B. Bruya (Ed.), *Effortless attention: A new perspective in the cognitive science of attention and action* (pp. 103–120). MIT Press. 10.7551/mitpress/9780262013840.003.0005

[CR42] Pfister R (2019). Effect-based action control with body-related effects: Implications for empirical approaches to ideomotor action control. Psychological Review.

[CR43] Pfister R, Janczyk M (2013). Confidence intervals for two sample means: Calculation, interpretation, and a few simple rules. Advances in Cognitive Psychology.

[CR44] Pfister, R., & Janczyk, M. (2020). *schoRsch: Tools for analyzing factorial experiments* (R Package Version 1.9.1) [Statistical software]. Retrieved from https://CRAN.R-project.org/package=schoRsch. Accessed 2 Nov 2022.

[CR45] Pfister R, Kunde W (2013). Dissecting the response in response–effect compatibility. Experimental Brain Research.

[CR46] Potts CA, Carlson RA (2019). Control used and control felt: Two sides of the agency coin. Attention, Perception, & Psychophysics.

[CR47] Ramsey R, Cumming J, Eastough D, Edwards MG (2010). Incongruent imagery interferes with action initiation. Brain and Cognition.

[CR48] R Core Team. (2020). *R: A language and environment for statistical computing.* R Foundation for Statistical Computing. Retrieved from https://www.R-project.org/. Accessed 2 Nov 2022.

[CR49] Richardson B, Pfister R, Fournier LR (2020). Free-choice and forced-choice actions: Shared representations and conservation of cognitive effort. Attention, Perception, & Psychophysics.

[CR50] Rosenbaum DA, Fournier LR, Levy-Tzedek S, McBride DM, Rosenthal R, Sauerberger K, VonderHaar RL, Wasserman EA, Zentall TR (2019). Sooner rather than later: Precrastination rather than procrastination. Current Directions in Psychological Science.

[CR51] Rosenbaum DA, Gong L, Potts CA (2014). Pre-crastination: Hastening subgoal completion at the expense of extra physical effort. Psychological Science.

[CR52] Schwarz KA, Pfister R, Wirth R, Kunde W (2018). Dissociating action–effect activation and effect-based response selection. Acta Psychologica.

[CR53] Shin YK, Proctor RW, Capaldi EJ (2010). A review of contemporary ideomotor theory. Psychological Bulletin.

[CR54] Shin YK, Proctor RW (2012). Testing boundary conditions of the ideomotor hypothesis using a delayed response task. Acta Psychologica.

[CR55] Sidarus N, Chambon V, Haggard P (2013). Priming of actions increases sense of control over unexpected outcomes. Consciousness and Cognition.

[CR56] Sidarus, N., Vuorre, M., Metcalfe, J., & Haggard, P. (2017). Investigating the prospective sense of agency: Effects of processing fluency, stimulus ambiguity, and response conflict. *Frontiers in Psychology*, *8*. 10.3389/fpsyg.2017.00545 10.3389/fpsyg.2017.00545 PMC5389984 28450839

[CR57] Tlauka M, McKenna FP (1998). Mental imagery yields stimulus–response compatibility. Acta Psychologica.

[CR58] Wickham, H. (2016). *ggplot2: Elegant Graphics for Data Analysis.* Springer.

[CR59] Wickham et al., (2019). Welcome to the tidyverse. *Journal of Open Source Software, 4*(43), Article 1686, 10.21105/joss.01686

[CR60] Wirth R, Pfister R, Brandes J, Kunde W (2016). Stroking me softly: Body-related effects in effect-based action control. Attention, Perception, & Psychophysics.

[CR61] Wolfensteller U, Ruge H (2011). On the timescale of stimulus-based action–effect learning. Quarterly Journal of Experimental Psychology.

