# Supplementary material for: Action control costs in task selection: Agents avoid actions with incompatible movement and effect features
Source: Atten Percept Psychophys. 2024 Mar 21;86(4):1330–41. doi: 10.3758/s13414-024-02863-0 (PMC11093875; doi:10.3758/s13414-024-02863-0)
Supplement: Supplementary file 1 — Supplementary file1 (DOCX 218 KB) [file 13414_2024_2863_MOESM1_ESM.docx]

Supplementary material for

**Action control costs in task selection: Agents avoid actions with incompatible movement and effect features**

**Analysis of the extended sample**

During the analyses reported in the main text, we applied strict rejection criteria to ensure high data quality and to filter out those participants who conformed to a rigid strategy when choosing between action options. However, we also performed the main analyses with a larger sample where we retained as many participants as possible. For the analysis of choice proportions, the only condition was that participants cannot commit a direction or speed error on more than 30 percent of the experimental trials. With this criterion, we only had to exclude one participant resulting in a sample of 92 participants (mean age: 24.60 years, age range: 19 – 30 years, male: 34, female: 58, all right-handed). In accordance with the results reported in the main text, the percentage of compatible choices was also substantially above chance level in this larger sample, *t*(91) = 5.18, *p* < .001, *d* = 0.54 (see Figure S1 and Table S2). This effect was stable across the experiment: The repeated-measures ANOVA did not reveal a significant effect of block (*F*(3, 273) = 1.23, *p* = .293, η_p_^2^ = .01) and choice percentage was significantly higher than chance throughout the experiment (Table S1).


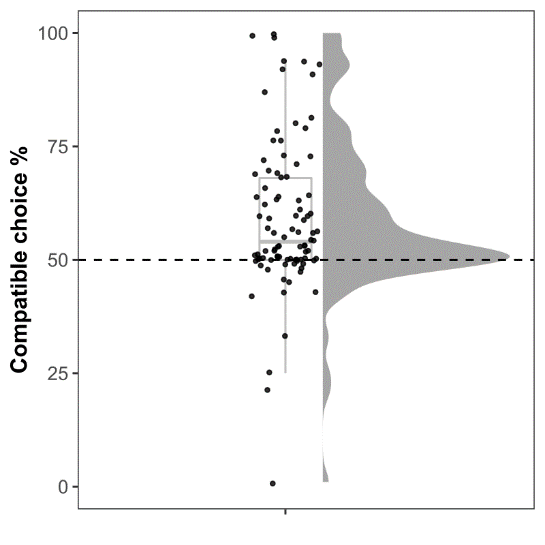


**Figure S1. Percentage of compatible choices for participants included in the extended sample.** Points represent participants’ individual choice percentage, accompanied by a boxplot showing the median (thick line), the interquartile range (IQR, box) and the 1.5 IQR interval from the upper and lower limits of the IQR (whiskers). A kernel density estimate is displayed next to the individual values.

| *Table S1. Percentage of compatible choices in the four experimental blocks (extended sample).* | | | |
| --- | --- | --- | --- |
|  | *M*  (percent) | *SD*  (percent) | Comparison to chance level^1^ |
| 1^st^ block | 58.39 | 17.68 | 4.55***  *0.47* |
| 2^nd^ block | 58.69 | 20.04 | 4.16***  *0.43* |
| 3^rd^ block | 58.08 | 20.41 | 3.80***  *0.40* |
| 4^th^ block | 61.13 | 20.79 | 5.13***  *0.54* |
| *** *p* < .001  ^1^ We report *t*-values with significance levels and effect sizes (in italics: Cohen’s *d_z_*) | | | |

For the analysis of error percentage, we excluded participants who consistently selected the same option throughout the whole experiment (retaining 89 participants for the analysis). Error percentage was higher in the incompatible compared to the compatible trials (*t*(88) = 2.12, *p* = .036, *d_z_* = 0.23). We found a weak but significant correlation between the difference of error percentages (error percentage in the incompatible trials minus error percentage in the compatible trials) and compatible choice percentage (*r*(87) = .23, *p* = .034). For the analysis of temporal action parameters, we also excluded participants who used a monitor with a frame rate below 50 Hz. As for the analyses in the main text, we excluded trials with reaction times or action durations 2.5 standard deviations above or below the mean RT for the given participant and action option (compatible, incompatible) as well as trials with direction or speed errors. Participants who had less than 5 trials remaining for either of the action options after applying these criteria were excluded from the analyses, resulting in a sample of 79 participants (mean age: 24.39 years, age range: 19 – 30 years, male: 30, female: 49, all right-handed). Replicating the results in the main text, reaction times were significantly faster in compatible, than in incompatible trials, *t*(78) = 3.14, *p* = .002, *d_z_* = 0.35. Additionally, the results indicated a weak positive correlation between reaction time advantage for compatible trials and percentage of compatible choices (i.e., participants who showed a larger compatibility effect in reaction times also tended to select the compatible action more often) that did not reach the significance level, however, *r*(77) = .196, *p* = .084. We did not observe significant effects in movement times, *t*(78) = 1.16, *p* = .250, *d* = 0.13, or in selection times, *t*(78) = 0.05, *p* = .961, *d* = 0.01, again mirroring the pattern of the main analyses.

| *Table S2. Choice and error percentage, reaction time and movement time for compatible and incompatible trials (extended sample). Each pair of means is accompanied by the corresponding standard error of paired differences (SE_PD_; Pfister & Janczyk, 2013)* | | | | | | | | | | | | |
| --- | --- | --- | --- | --- | --- | --- | --- | --- | --- | --- | --- | --- |
|  | Choice percentage (%) | | | Error  percentage (%) | | | Reaction time (ms) | | | Movement time (ms) | | |
|  | *M* | *SD* | *SE_PD_* | *M* | *SD* | *SE_PD_* | *M* | *SD* | *SE_PD_* | *M* | *SD* | *SE_PD_* |
| Compatible | 59.08 | 16.81 | 3.50 | 3.77 | 4.85 | 0.64 | 704 | 184 | 9.18 | 68 | 60 | 27.25 |
| Incompatible | 40.92 | 16.81 |  | 5.13 | 7.08 |  | 733 | 200 |  | 99 | 253 |  |

In the extended sample of 89 participants (participants who consistently selected the same option throughout the whole experiment were excluded), there were 33 participants who had a higher error rate in the compatible condition, 13 participants who had identical error rates in the two conditions and 43 participants who committed errors at a higher rate in the incompatible condition. In the subsample consisting of participants with equal error rates in the two conditions or higher error rate in the compatible condition, a similar preference of the compatible option (*M* = 57.09, *SD* = 15.13) emerged as in the full extended sample, *t*(45) = 3.18, *p* = .003, *d* = 0.47. Similarly to the results reported in the main text, in the remaining part of the sample (i.e., participants who committed errors at a higher rate in the incompatible condition), the preference for the compatible option was slightly more pronounced (*t*(42) = 4.98, *p* < .001, *d_z_* = 0.78). However, the difference in compatible choice percentage was not significantly different between the two subsamples (*t*(87) = 1.54, *p* = .126, *d* = 0.33).

**Mixed-effect model analysis of choices and reaction times**

As an exploratory analysis, we submitted location choice (top, bottom) to a generalized linear mixed model with subject as a random effect and location of compatible option (top, bottom) and previous choice location^[[1]](#footnote-1)^ (top, bottom) as fixed effects. Random by-subject slope for previous choice location was included in the model. Further predictors were included based on a data-driven approach: In a stepwise manner, we added further fixed effect factors—color of stimulus on the top (green, blue) and reaction time on previous trial (transformed to z-scores)—to the model. These additional effects were only retained if they improved the model substantially, as indicated by the Akaike information criterion (AIC). Estimates are reported for the following model:

**location choice ~ location of compatible option * previous choice location + (1 + previous choice location | subject)**

To examine the influence of preceding choices on reaction times, we also analyzed reaction times with a GLMM that included compatibility (compatible, incompatible) and relation to preceding choice (repetition, change) as fixed effect predictors and subject as a random effect factor (with a random intercept and random slopes for compatibility and relation to preceding choice effects as well as for the compatibility × relation to preceding choice interaction). This initial model was stepwise augmented by additional fixed effect factors: movement direction, previous movement direction, movement direction change (i.e., movement direction × previous movement direction interaction), stimulus location (top, bottom), stimulus color (green, blue) and reaction time on previous trial (transformed to *z-*scores). From these additional effects, only those were retained for the final GLMM that improved the model substantially as indicated by the AIC. Estimates are reported for the following model:

**rt ~** **compatibility * relation to previous choice + movement direction change + stimulus location + reaction time on previous trial + (1 + compatibility * relation to previous choice | subject)**

Due to skewed reaction time distributions, we used a GLMM (with an identity link function) that assumes an Inverse Gaussian distribution (Lo & Andrews, 2015). For all GLMMs, significance of fixed effects was examined by likelihood ratio tests of the full model and a reduced model without the effect in question (comparison of nested models).

Mixed effects model analyses were aimed at assessing the influence of preceding trials on performance, thus, beside applying the same rejection criteria as in analyses reported in the main text, the first trial of each block was also omitted from these analyses. Since errors can substantially affect reaction times in the subsequent trial (i.e., post-error slowing), trials following direction or speed errors were also excluded when applying GLMM to reaction times.

In keeping with the primary analyses, the GLMM of the location choices indicated a substantial influence of location for compatible choice (χ^2^(1) = 25.160, *p* < .001, Figure S2): The odds of choosing the object on the top is ca. 2.122 times larger if the wagon on the top is following the direction of the mouse movement than if it is moving in the opposite direction. The effect of previous choice location and the location for compatible choice × previous choice location interaction, however, did not improve the model fit significantly (see Table S3).


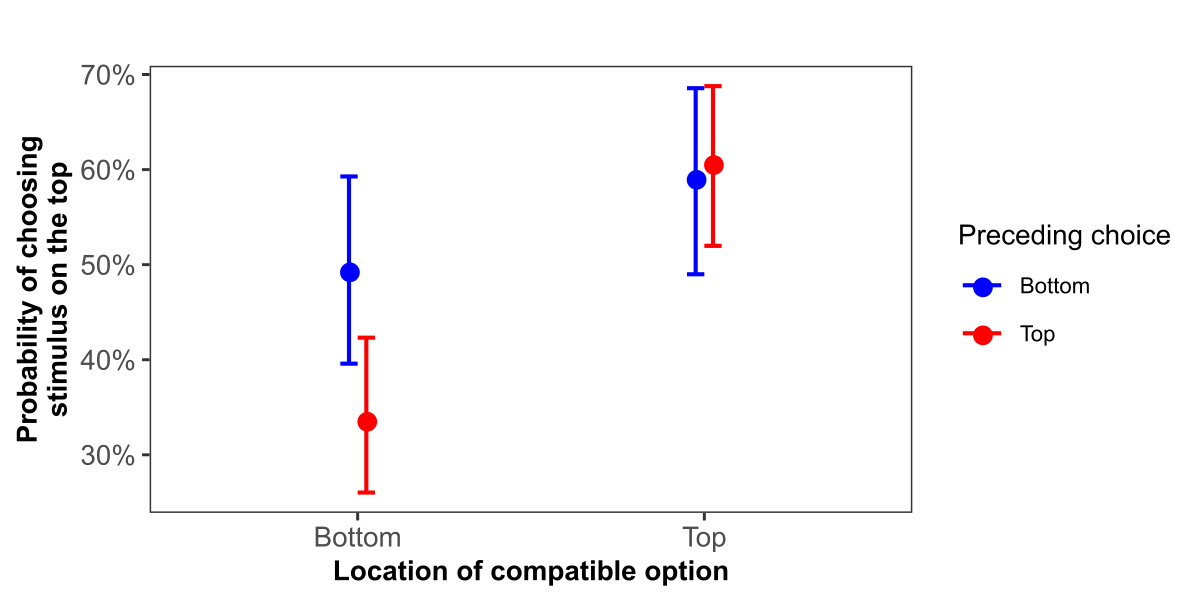


**Figure S2. Predicted probability of choosing the stimulus at the top of the screen.** Predictions are displayed as the function of compatible option location (top or bottom) and location of the preceding choice (top or bottom). Points represent estimated marginal means, transformed to probability scale, whiskers show 95% confidence intervals for the predicted values.

| *Table S3. Estimated coefficients for the mixed model fitted to the choice locations* | | | | | | |  |
| --- | --- | --- | --- | --- | --- | --- | --- |
| *Predictors* | *Estimate*^1^ | *SE* | *CI* | *z-value* | *p* | *Random effect*  *SD* | |
| Intercept | 0.03 | 0.07 | -0.11– 0.16 | 0.38 | .701 | 0.43 | |
| Location of compatible option (Top) (L) | 0.75 | 0.13 | 0.49 – 1.02 | 5.58 | **<.001** | **--** | |
| Pevious choice location (Top) (P) | -0.29 | 0.24 | -077 – 0.18 | -1.22 | .226 | 1.71 | |
| L:P | 0.72 | 0.48 | -023 – 1.67 | 1.49 | .141 | **--** | |
| Marginal R^2^ / Conditional R^2^ | | | .041 / .249 | | | |  |
| ^1^Untransformed estimates (log odds ratios) are displayed. | | | | | | |  |

Confirming the results obtained with the *t-*test*,* the GLMM of the reaction times showed that actions were faster by ca. 29 ms when participants selected the object with the compatible movement (χ^2^(1) = 7.456, *p* = .006). The model also indicated that actions were executed faster (by ca. 28 ms), when participants repeated their choice from the previous trial (Figure S3; see also Lelonkiewicz et al., 2020; Schonard et al., 2021). The predictors Movement direction change (faster response if the direction of the movement was the same as in the preceding trial), Stimulus location (faster response when the object on the top of the screen was selected), and Preceding reaction time (faster reaction times on the actual trial if the response on the previous trial was also fast) also improved model fit significantly (see Table S4).

| *Table S4. Estimated coefficients for the mixed-model fitted to the reaction times* | | | | | | |
| --- | --- | --- | --- | --- | --- | --- |
| *Predictors* | *Estimate*^1^ | *SE* | *CI* | *t-value* | *p* | *Random effect SD* |
| Intercept | 0.73 | 0.03 | 0.68 – 0.79 | 25.81 | **<.001** | 0.07 |
| Compatibility (Compatible)(C) | -0.03 | 0.01 | -0.05 – -0.01 | -2.81 | **.006** | 0.03 |
| Relation to previous trial (Repetition) (R) | -0.03 | 0.01 | -0.04 – -0.01 | -3.47 | **<.001** | 0.02 |
| Movement direction change (Repetition) | -0.01 | <0.01 | -0.02 – -0.004 | -3.06 | **.002** | **--** |
| Stimulus location (Top) | -0.02 | 0.01 | -0.04 – 0.001 | -1.89 | .064 | **--** |
| Preceding RT (z-score) | 0.02 | <0.01 | 0.02 – 0.03 | 6.60 | **<.001** | **--** |
| C:R | -0.03 | 0.02 | -0.06 – 0.01 | -1.41 | .155 | 0.06 |
| Marginal R^2^ / Conditional R^2^ | | | .004 / .025 | | | |
| ^1^Note that RTs measured on the seconds scale were used in the model to keep the dependent variable in similar range as the standardized continuous predictor (preceding RT z-score). Thus, estimates in the table refer to increments in seconds. In Figure 3, however, predicted reaction time values are displayed on the milliseconds scale. | | | | | | |


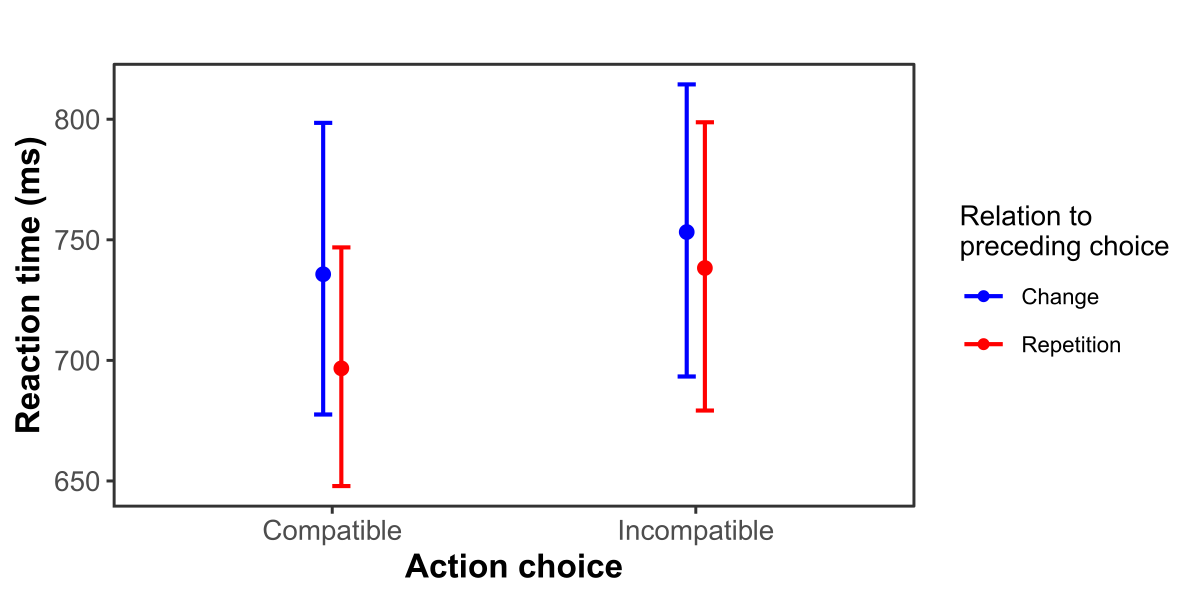


**Figure S3. Predicted reaction time values.** Points represent estimated marginal means for the Action choice and Relation to preceding choice factors, whiskers show 95% confidence intervals for the predicted values.

**Additional analyses of post-error switching**

The probability of changing the selected action option was not increased by an error on the preceding trial, contradicting the assumption underlying the post-error switching theory: The GLMM of action option change with preceding choice (compatible, incompatible) and error on preceding trial (error, no error) as fixed effect predictors and subject as a random effect (see Table S5 for details of the model) indicated that the probability of action option change decreases after error trials (Figure S4). This switch-preventing influence of error trials was more pronounced for compatible choices, as indicated by the preceding choice × preceding error interaction (Table S5).

| *Table S5. Estimated coefficients for the mixed-model fitted to action option chane* | | | | | | |
| --- | --- | --- | --- | --- | --- | --- |
| Model: **option change ~ preceding error * preceding choice + (1 + preceding error \| sub)** | | | | | | |
| *Predictors* | *Estimate*^1^ | *SE* | *CI* | *z-value* | *p* | *Random effect SD* |
| Intercept | -0.43 | 0.14 | -0.71 – -0.15 | -2.97 | **.003** | 0.48 |
| Preceding error (error trial) (E) | -1.06 | 0.26 | -1.56 – -0.55 | -4.08 | **<.001** | 1.04 |
| Preceding choice (compatible) (C) | -1.04 | 0.20 | -1.42 – -0.65 | -5.29 | **<.001** | **--** |
| E:C | -0.77 | 0.39 | -1.54 – -0.01 | -1.97 | **.040** | **--** |
| Marginal R^2^ / Conditional R^2^ | | | .036 / .227 | | | |
| ^1^Untransformed estimates (log odds ratios) are displayed. | | | | | | |


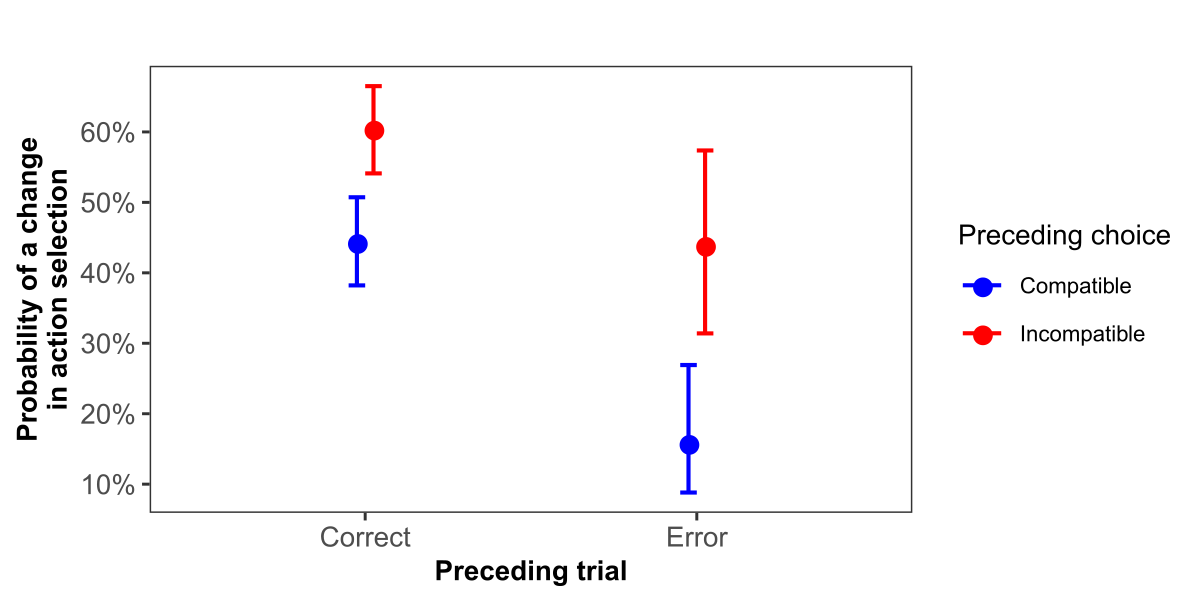


**Figure S4. Predicted probability of changing the action option selected in the preceding trial.** Predictions are displayed as the function of preceding error (correct trial or error trial) and preceding choice (compatible or incompatible). Points represent estimated marginal means, transformed to probability scale, whiskers show 95% confidence intervals for the predicted values.

The larger switch-preventing effect of compatible trials could suggest that participants who commit more errors in compatible trials, switch away less often from the compatible option, which would result in a higher compatible choice tendency. The (marginally) lower compatible choice percentage in the subset of participants who commit errors at a higher rate in compatible trials (or have the same error percentage with both action options), however, does not fit into this picture. Our results might suggest that errors factor into the costs associated with the action options: That is, participants chose options where they are less probable to commit an error. However, this aversion against the error-prone option is not realized immediately after the error, which could be explained by the complexity of the task structure: In the current design, error correction might be easier when rules governing the error trial and the subsequent trial are identical. Thus, participants might stick with the same option, even after an error, until they feel confident about executing the task. It is also possible, that participants are motivated by error feedback to re-select the same option after they committed an error (Stephens, 1934). However, in the long run, the probability of errors might be taken into account and participants seem to prefer options where they are less likely to make mistakes.

**Sensitivity to errors in trials with incompatible choice**

The interpretation of directional errors and the adjustments that have to be made in response to them are quite straightforward when action and effect is compatible. Things get more complicated, however, when the incompatible option is selected: If participant execute a left swipe in response to a right directional cue, they will receive feedback that the direction of the movement was wrong. However, the effect that they saw on the screen moved in the opposite direction as the hand, thus, its movement coincided with the direction signaled by the cue (to the right). As a consequence, error feedback might not only inhibit the association between the cue and leftward movement but also the association between the cue and rightward movement. To limit the increased cognitive demands associated with error monitoring and error correction in incompatible trials, participants might resort to avoiding the incompatible option, which could explain the higher proportion of compatible choices. Similarly to other explanations described in the main text, this interpretation of the results is also based on the assumption that preference of the compatible option relies on the larger cognitive effort associated with the incompatible option. However, in this case, this anticipatory effect would be specific to the experimental design and the relevance of our finding would be limited to situations with error feedback.

To explore whether our data support the above interpretation, we selected trials that were performed by participants before their first directional error. If preference of compatible choices is indeed determined by the cognitive demands of error feedback processing and error correction, the higher proportion of compatible choices should not be present in this subset of the data. We only included participants who had at least 10 experimental trials before the first error. (Participants who made an error during the practice phase were automatically excluded.) 18 participants remained for the analysis. For them, we calculated the proportion of compatible choices, and compared it to chance level (50 percent) with a one-sample *t*-test.

Even in the pre-error stage of the task, participants chose the compatible option with a frequency above chance level (*M* = 58.34%, *SD* = 9.59%), *t*(17) = 3.69, *p* = .002, *d* = 0.87. To have a larger sample size, we also conducted the same analysis on the extended sample. In this case, 29 participants remained who had at least 10 experimental trials in the pre-error stage. Results were very similar: On average, participants selected the compatible option on 58.95% (*SD* = 12.69%) of the trials, which is significantly above chance level, *t*(28) = 3.80, *p* = .001, *d* = 0.71.

Participants selected the action option with the compatible effect more often than the incompatible option even before committing any directional errors, which indicates that the preference of compatible action-effect relations is not dependent on experience about the difficulty of error feedback processing and error correction.

**References**

Kiesel, A., Steinhauser, M., Wendt, M., Falkenstein, M., Jost, K., Philipp, A. M., & Koch, I. (2010). Control and interference in task switching—A review. *Psychological Bulletin*, *136*(5), 849–874. <https://doi.org/10.1037/a0019842>

Kool, W., McGuire, J. T., Rosen, Z. B., & Botvinick, M. M. (2010). Decision making and the avoidance of cognitive demand. *Journal of Experimental Psychology: General*, *139*(4), 665–682. <https://doi.org/10.1037/a0020198>

Lelonkiewicz, J. R., Gambi, C., Weller, L., & Pfister, R. (2020). Action–effect anticipation and temporal adaptation in social interactions. *Journal of Experimental Psychology: Human Perception and Performance*, *46*(4), 335–349. <https://doi.org/10.1037/xhp0000717>

Lo, S., & Andrews, S. (2015). To transform or not to transform: Using generalized linear mixed models to analyse reaction time data. *Frontiers in Psychology*, *6*. <https://doi.org/10.3389/fpsyg.2015.01171>

Monsell, S. (2003). Task switching. *Trends in Cognitive Sciences*, 7, 134–140. <https://doi.org/10.1016/S1364-6613(03)00028-7>

Schonard, C., Proctor, R. W., Xiong, A., Janczyk, M. (2021). Examination of a response–effect compatibility task with continuous mouse movements: Free- versus forced-choice tasks and sequential modulations. *The American Journal of Psychology, 134*(4), 415–439. <https://doi.org/10.5406/amerjpsyc.134.4.0415>

Stephens, J. M. (1934). The influence of punishment on learning. *Journal of Experimental Psychology*, *17*(4), 536–555. <https://doi.org/10.1037/h0072035>

1. As part of the exploratory analyses, we examined the effect of previous choice since in switching tasks, usually substantial differences are reported between switch and repeat trials (Kiesel et al., 2010; Monsell, 2003), and switch related costs have been used previously to operationalize cognitive demand (Kool et al., 2010). We expected that switch costs might be substantial in the current study, where switching results in a partial change in the direction feature associated with the cue (identical action direction but different effect direction) and, thus, in addition to compatibility-related effects this could be an important factor in choices that are based on cognitive control demands. [↑](#footnote-ref-1)
